# Spontaneous Regression of Non-small Cell Lung Cancer: A Case Report and Literature Review

**DOI:** 10.7759/cureus.6639

**Published:** 2020-01-12

**Authors:** Ashley Shatola, Ky Nam Nguyen, Elham Kamangar, Megan E Daly

**Affiliations:** 1 Radiation Oncology, University of California Davis School of Medicine, Sacramento, USA; 2 Radiation Oncology, University of California Davis Comprehensive Cancer Center, Sacramento, USA; 3 Pathology, University of California, Sacramento, USA

**Keywords:** lung cancer, spontaneous regression

## Abstract

Spontaneous cancer regression is rare, and particularly rare for non-small cell lung cancer (NSCLC). The pathogenesis of spontaneous regressions is poorly understood, but of interest to many patients and providers. The infrequency of spontaneous regression makes it a challenging phenomenon to understand and study. We present a case of biopsy-proven NSCLC in a 73-year-old man that regressed without treatment.

## Introduction

Spontaneous regression is defined as the complete or partial resolution of a malignant lesion without appropriate treatment [1], either permanently or temporarily. Spontaneous tumor regression is an extremely uncommon phenomenon occurring in fewer than one in one hundred thousand cancers [2]. Spontaneous regression has been most commonly reported in renal cell carcinoma, non-Hodgkin’s lymphoma, malignant melanoma, chronic lymphoid leukemia, and neuroblastoma [3]. It remains unclear why certain cancers spontaneously regress more frequently. One hypothesis is that spontaneous tumor regression is a result of an immune system process associated with the tumor's microenvironment and oncogenic expression [4].

We present a case of a man who experienced spontaneous regression of biopsy-proven non-small cell lung cancer (NSCLC) that markedly regressed over a six-month period without intervention and is now 14 months post-biopsy without evidence of active disease.

## Case presentation

**Figure 1:**
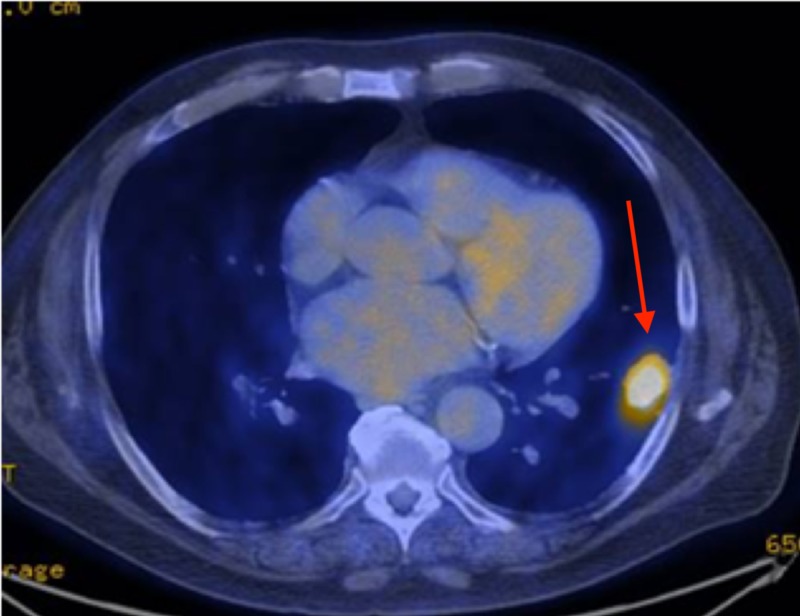
Axial fused PET/CT images taken pre-biopsy confirmed a solid nodule measuring 3.2 x 2.2 cm with SUV max of 14.1 PET/CT: positron emission tomography/computed tomography; SUV: standardized uptake value.

A 73-year-old Caucasian man with a 50 pack-year smoking history and a history of low grade, non-invasive bladder cancer treated with intravesicular Bacillus Calmette-Guerin (BCG) presented with worsening shortness of breath and dyspnea on exertion. The patient had no known history of autoimmune disease. Physical examination was unremarkable with clear lung sound, and no signs of clubbing or cyanosis. At the time of presentation, a C-reactive protein (CRP) and sedimentation rate (ESR) were ordered. Both were slightly elevated, the CRP was 1.0 mg/dL and ESR 24 mg/dL. A chest computed tomography (CT) was obtained for further evaluation, which revealed a 3.2 x 2.5 cm spiculated solid mass in the left lower lobe of the lung. Positron emission tomography (PET)/CT completed three weeks later confirmed a hypermetabolic solid nodule measuring 3.2 x 2.2 cm with a standardized uptake value (SUV) max of 14.1 consistent with primary lung cancer, with no evidence of metastatic disease (Figure 1).

Several borderline-enlarged mediastinal nodes with SUV ranging from 3.3-3.6 were also noted. Paratracheal level 2 and 4 nodes measured up to 1.2 cm, with a right hilar level 10 node measuring up to 1.3 cm.

**Figure 2:**
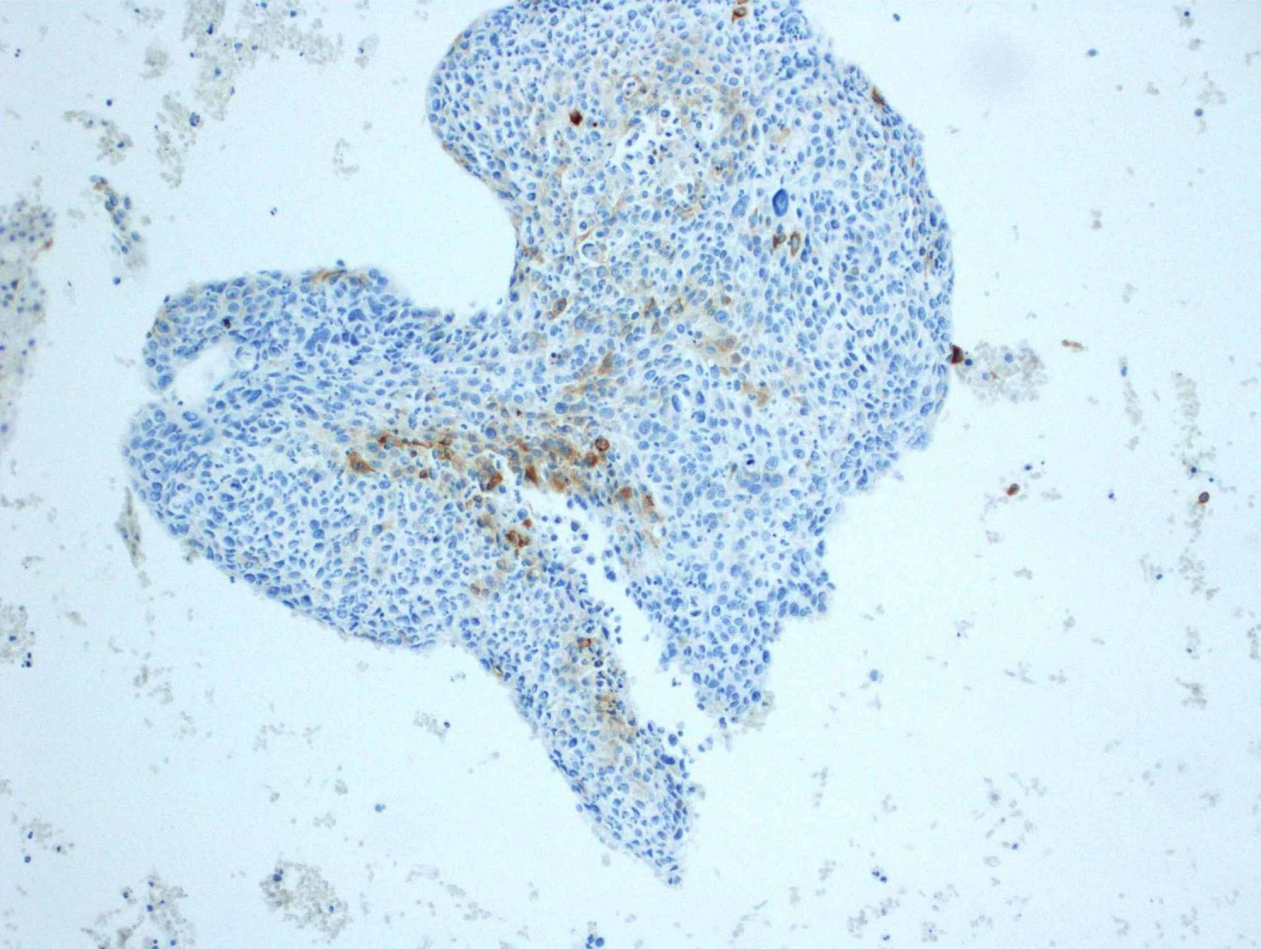
Focally positive Ber-EP4 immunohistochemistry stain

**Figure 3:**
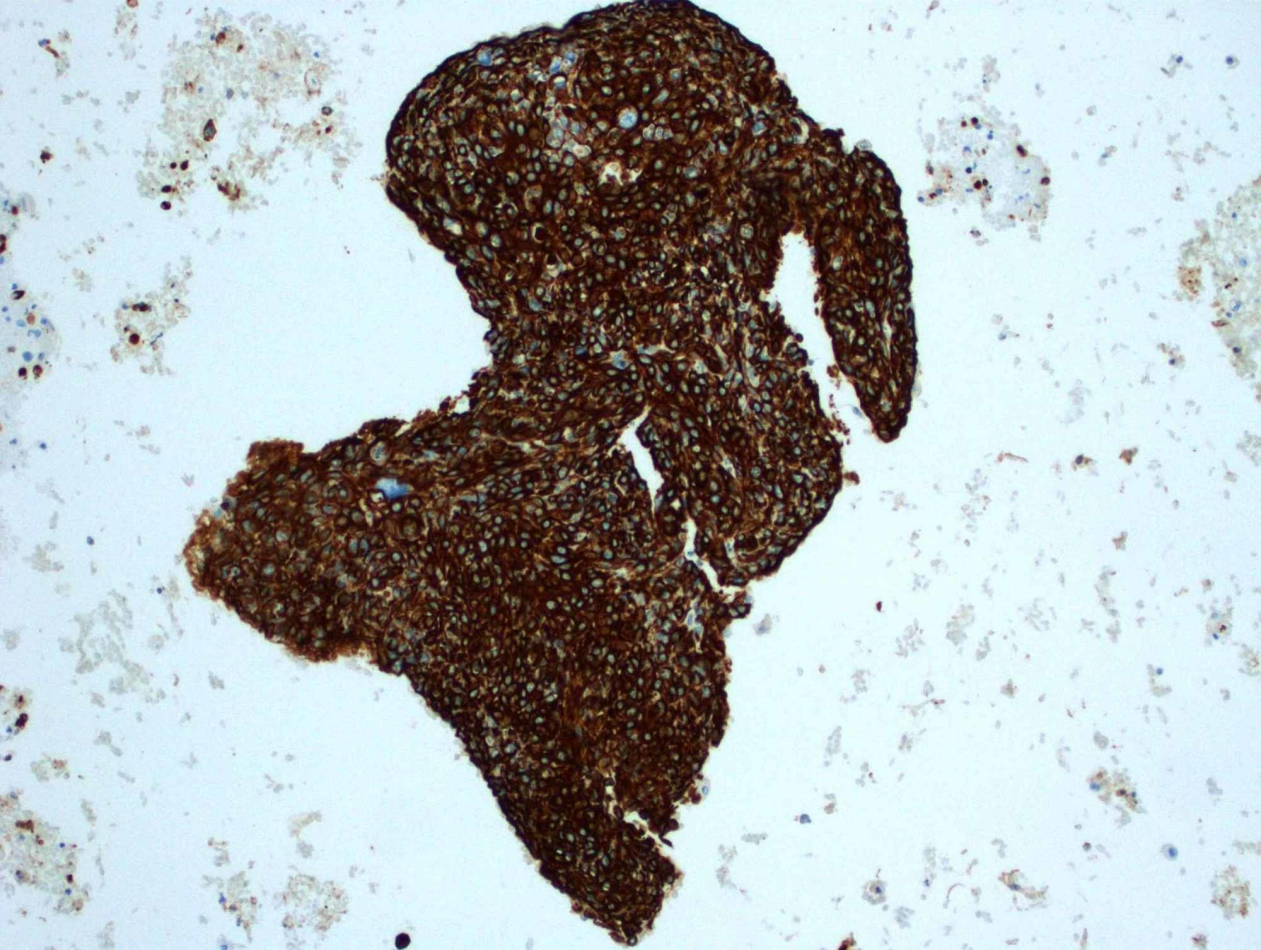
Positive stain for cytokeratin 5/6 expression

**Figure 4:**
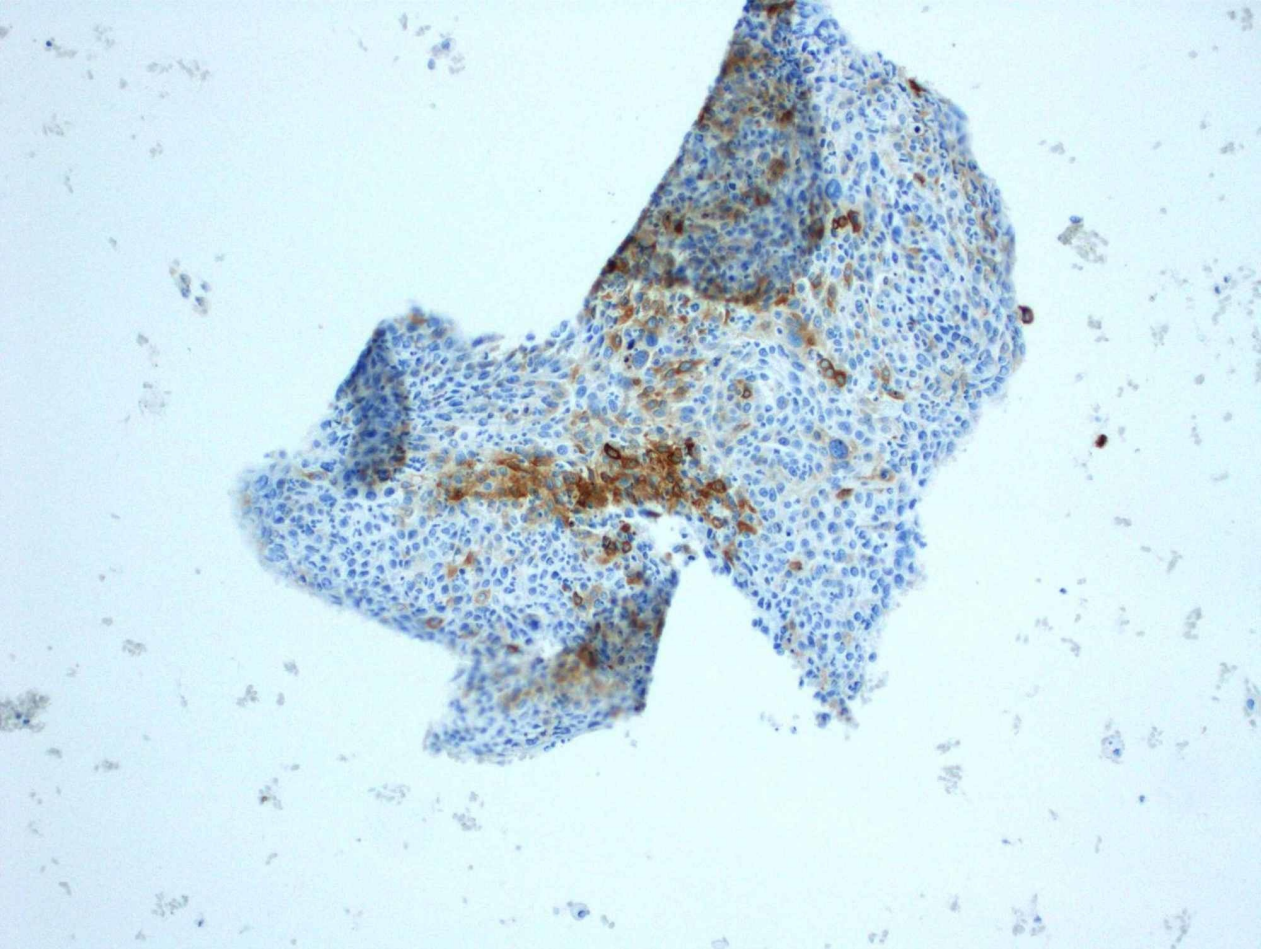
Focally positive epithelial membrane antigen stain

**Figure 5:**
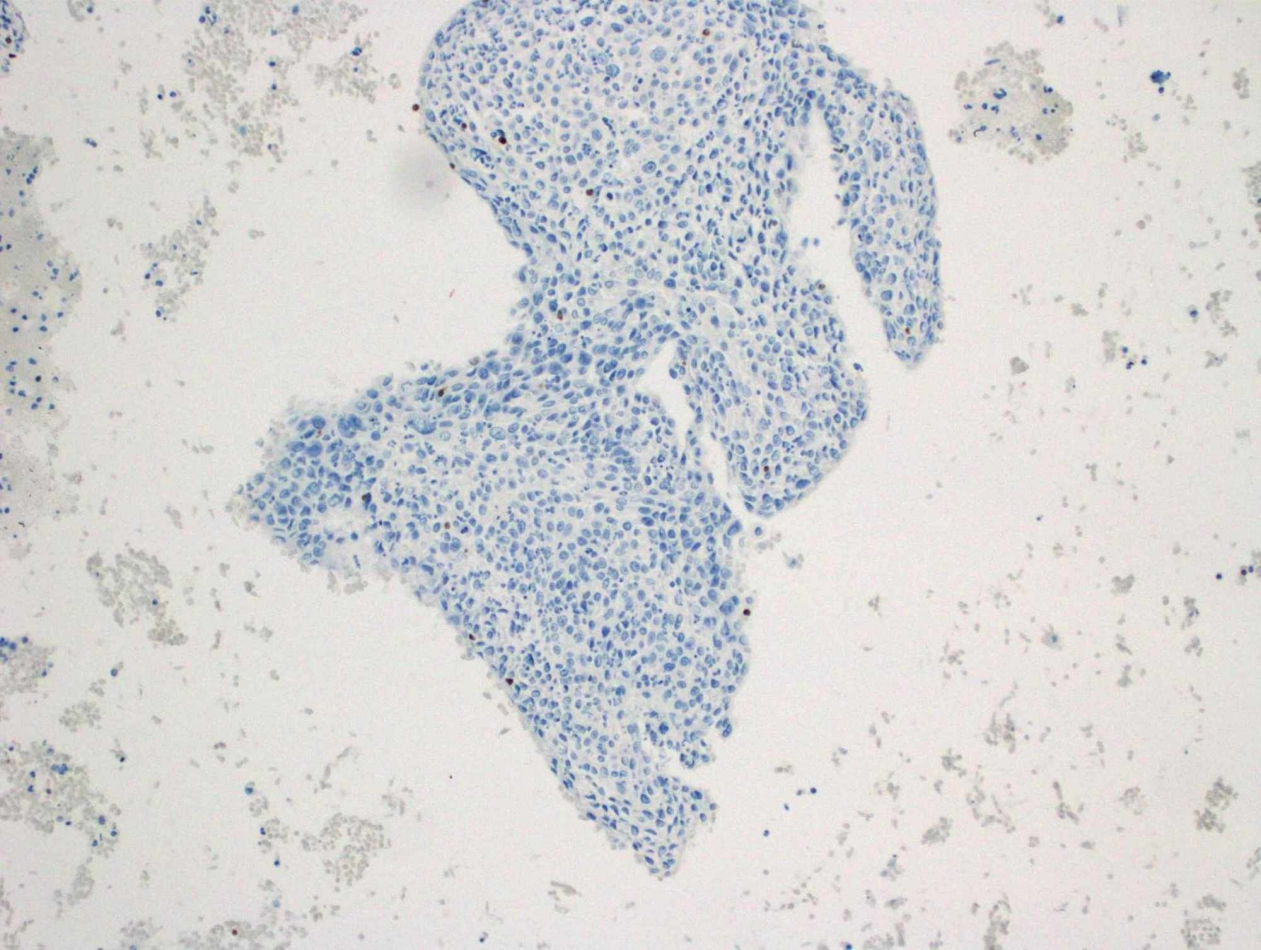
Negative nuclear staining for GATA3

**Figure 6:**
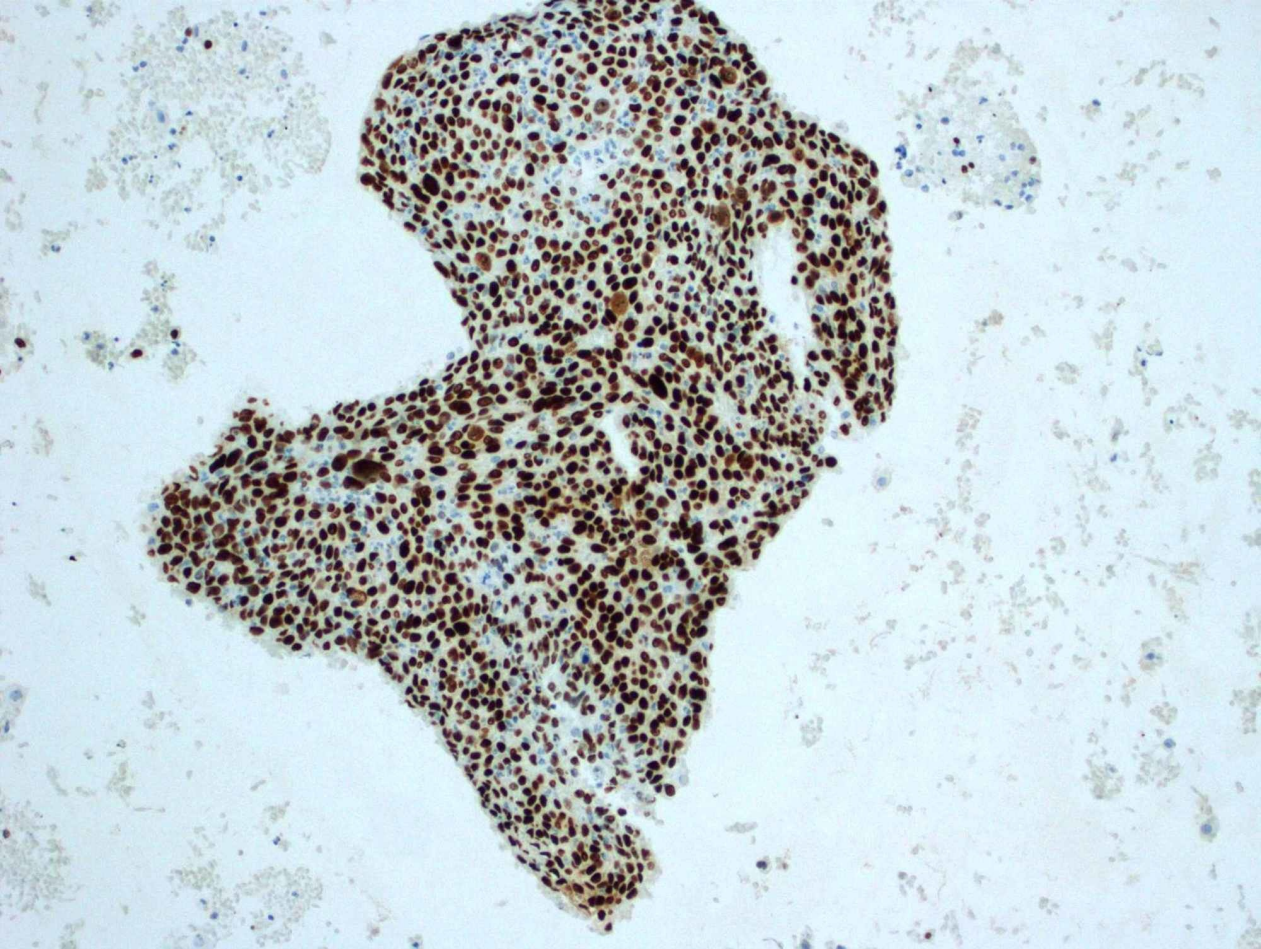
Positive nuclear staining for p63

**Figure 7:**
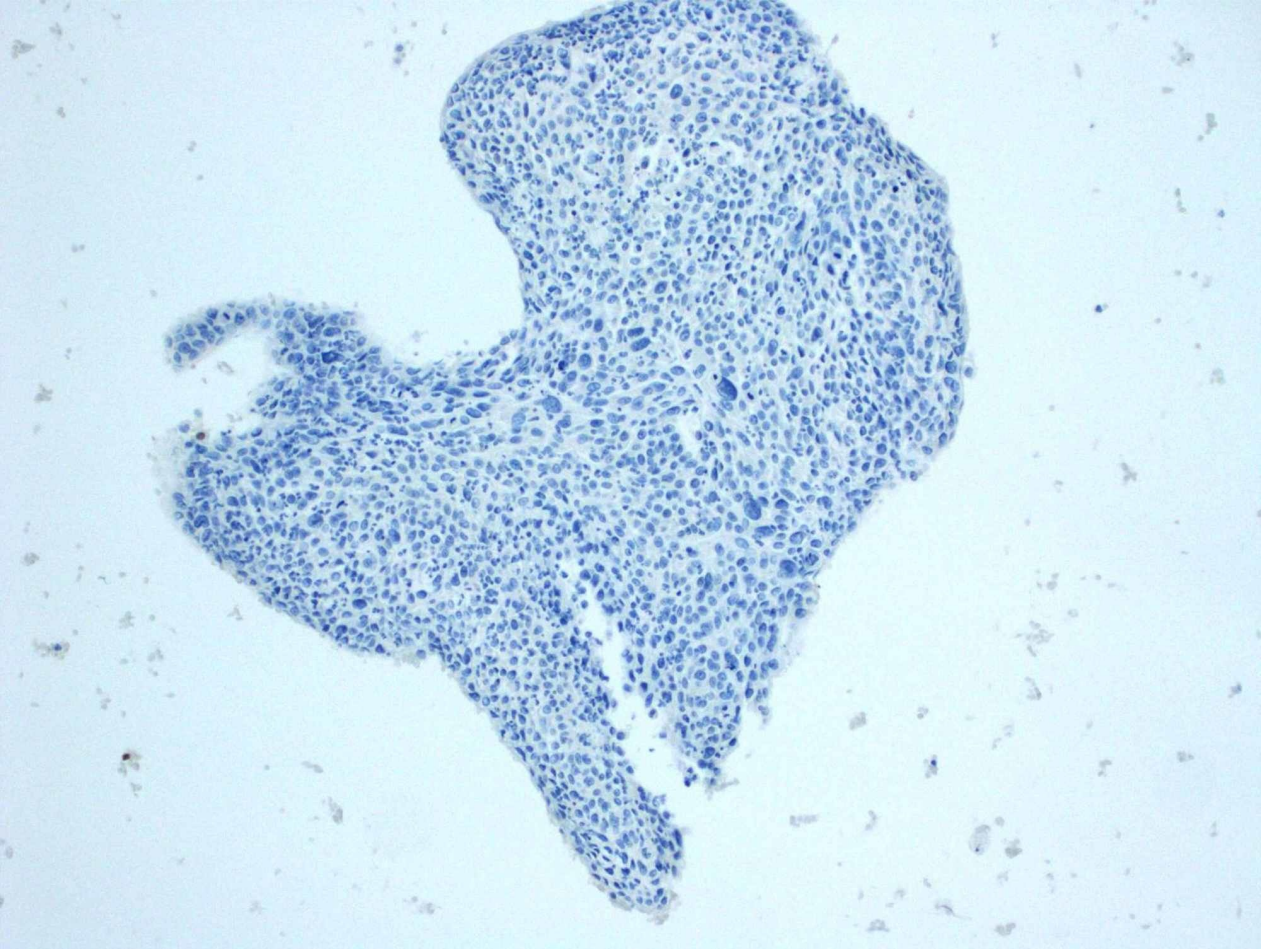
Negative Immunohistochemical staining for thyroid transcription factor-1 (TTF-1)

**Figure 8:**
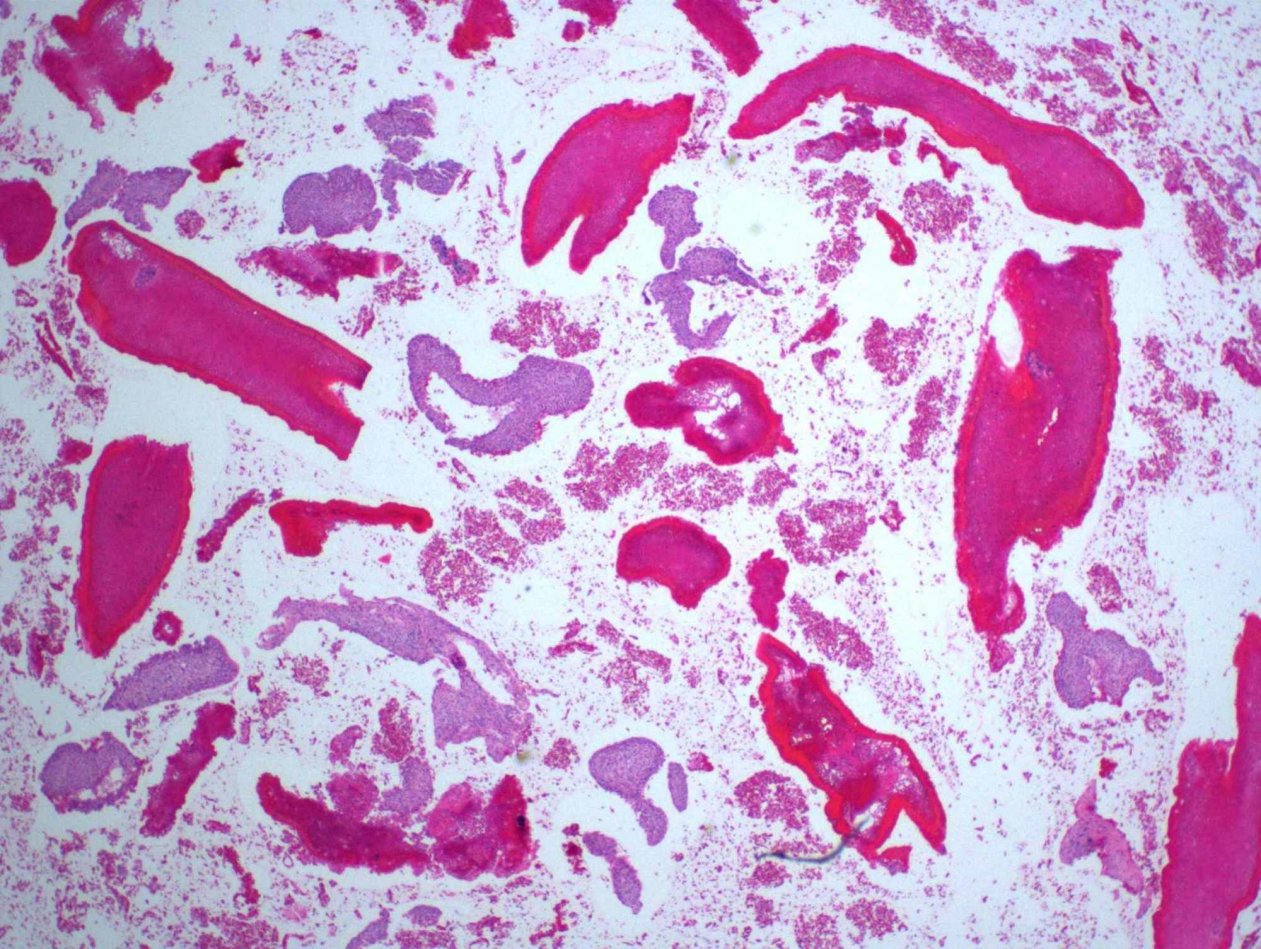
Hematoxylin and eosin stain

**Figure 9:**
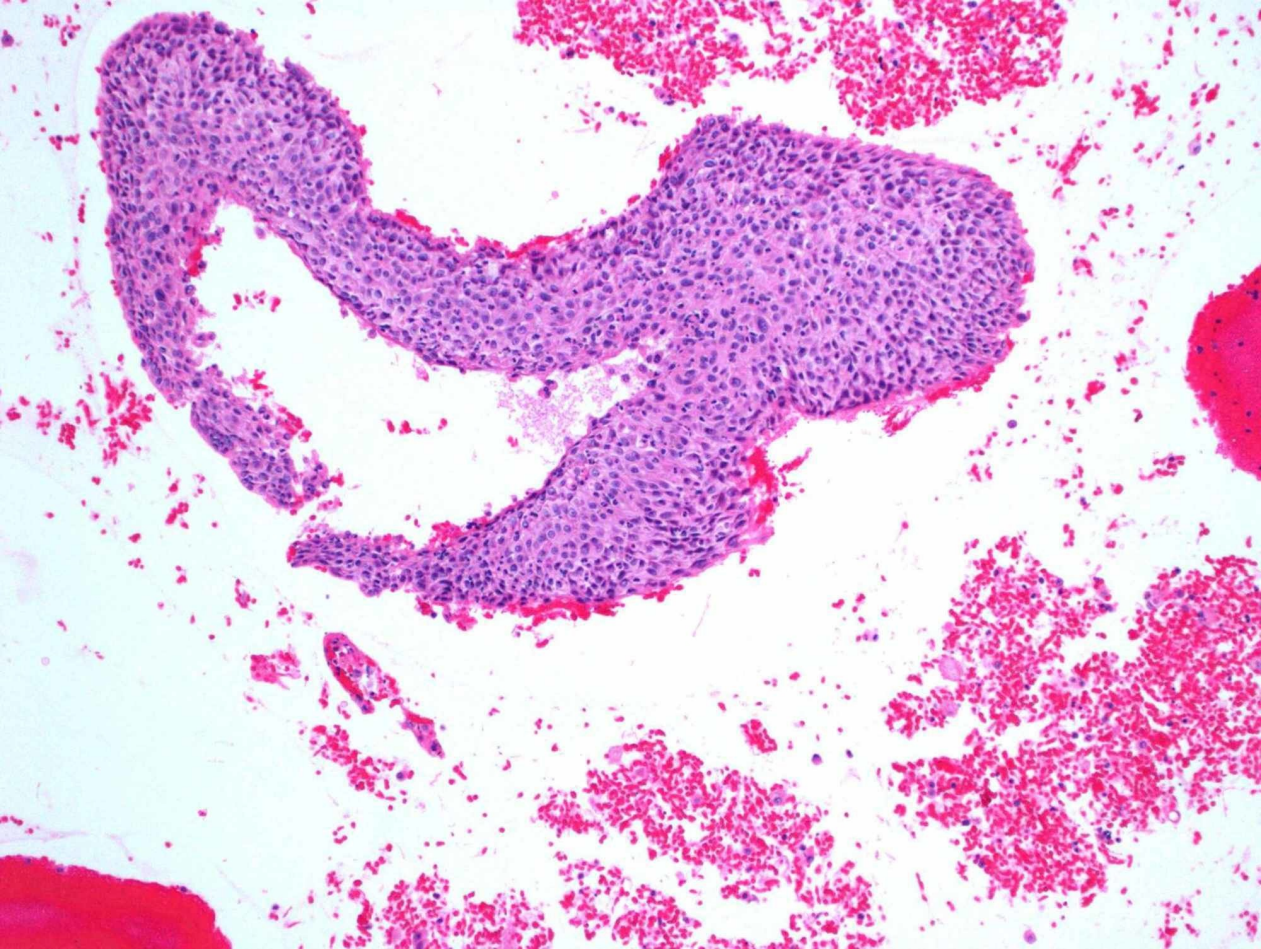
High power hematoxylin and eosin stain

Subsequent CT-guided fine needle aspirate using a 19-gauge thin-walled needle of the left lower lobe mass was performed, retrieving three aspirates. Cytology revealed large and small groups of neoplastic cells with enlarged and hyperchromatic nuclei, irregular nuclear membranes, some with small nucleoli and small amount of cytoplasm. On immunohistochemistry, cells were cytokeratin (CK)5/6 positive, epithelial membrane antigen (EMA) focally positive, Ber-EP4 very focally positive, GATA-3 negative, P63 positive and thyroid transcription factor 1 (TTF-1) negative (Figures 2-9). Overall the results were consistent with keratinizing squamous cell carcinoma. The pathology was reviewed in a multidisciplinary tumor board with an additional pathologist present who concurred with the diagnosis.

There were no post-biopsy lung changes or complications such as pneumothorax, pneumonitis, or bleeding. Of note, he completed a six-week course of weekly BCG instillations for non-invasive bladder cancer approximately one month prior to lung biopsy. A CRP obtained at this time point was mildly elevated at 1.0 (reference range: 0.1-0.8).

The patient was evaluated in both the thoracic surgery and radiation oncology clinics, with discussion of both surgical resection and stereotactic radiation as treatment options. Pulmonary function tests suggested sufficient pulmonary reserve to tolerate lobectomy. Endobronchial ultrasound (EBUS) with biopsies was recommended to assess borderline enlarged mediastinal nodes prior to a final treatment decision. Subsequently, there was a significant delay in completing the planned EBUS with biopsies related to patient challenges with transportation to and from medical appointments. The follow-up CT chest was obtained for re-staging purposes, now 10 weeks post-biopsy. The repeat CT chest revealed a marked decrease in the size of the left lower lobe lung mass from the most recent 3.2 cm to 2.2 cm.

After extensive discussion with the patient, and additional multidisciplinary tumor board review, the patient and treating physicians elected for short-interval follow-up imaging in three months in lieu of treatment.

**Figure 10:**
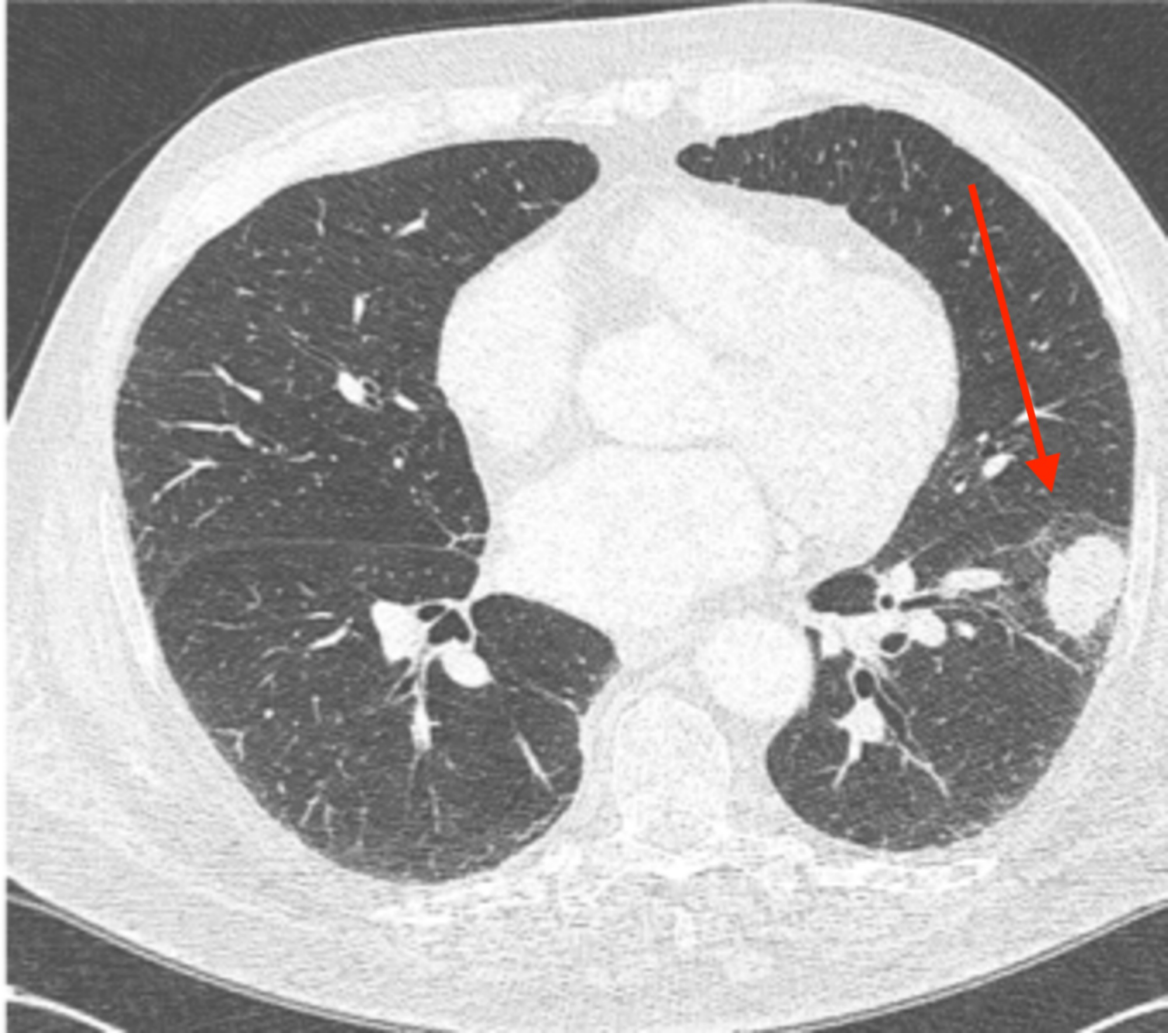
The axial slice from the baseline computed tomography (CT) chest is shown measuring 3.2 x 2.2 cm

**Figure 11:**
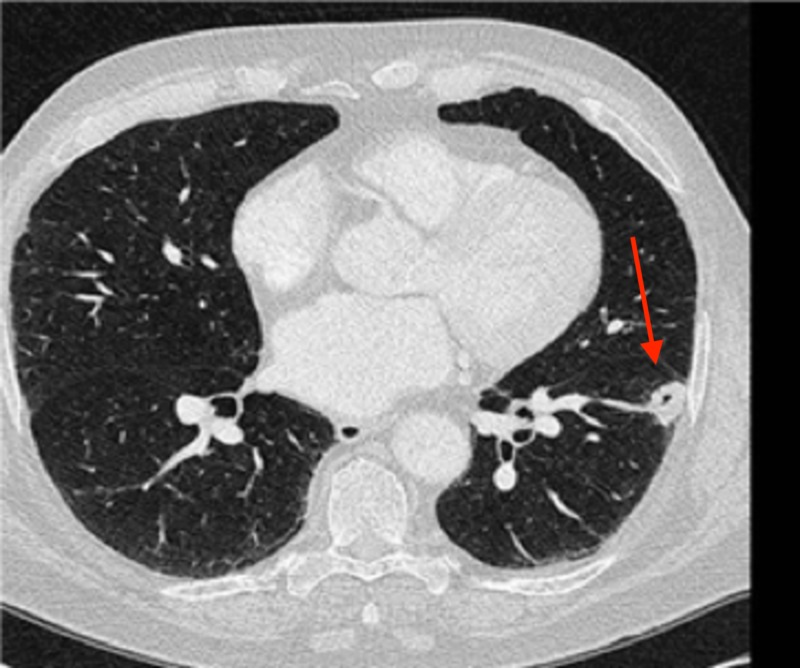
The regression of the untreated left lower lobe lung tumors is shown, with axial slice from the baseline computed tomography (CT) chest shown in Figure 1; an axial slide from three months later revealed a 2.2 x 1.6 cm tumor

**Figure 12:**
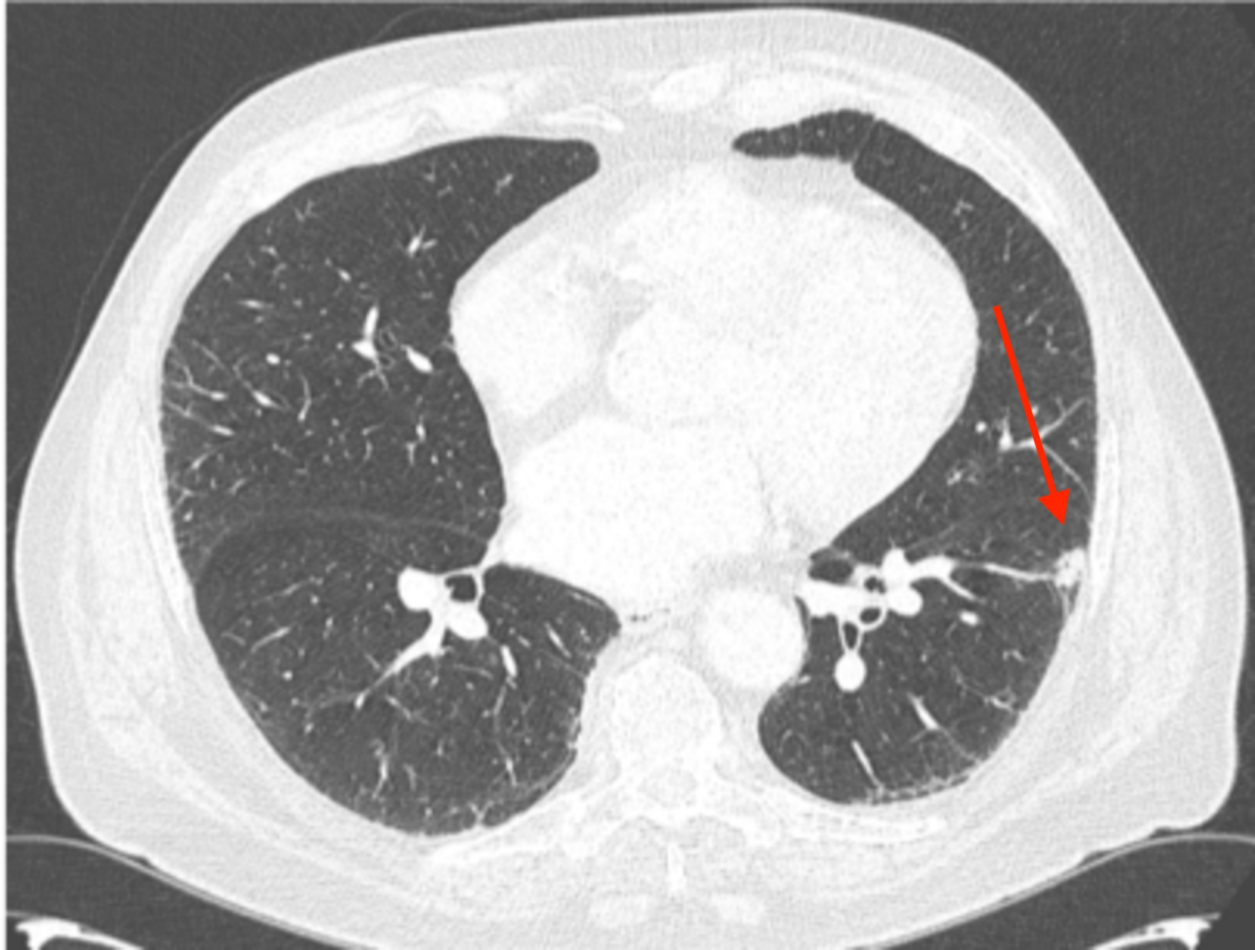
Three months later, the nodule measured 1.3 x 0.7 cm

**Figure 13:**
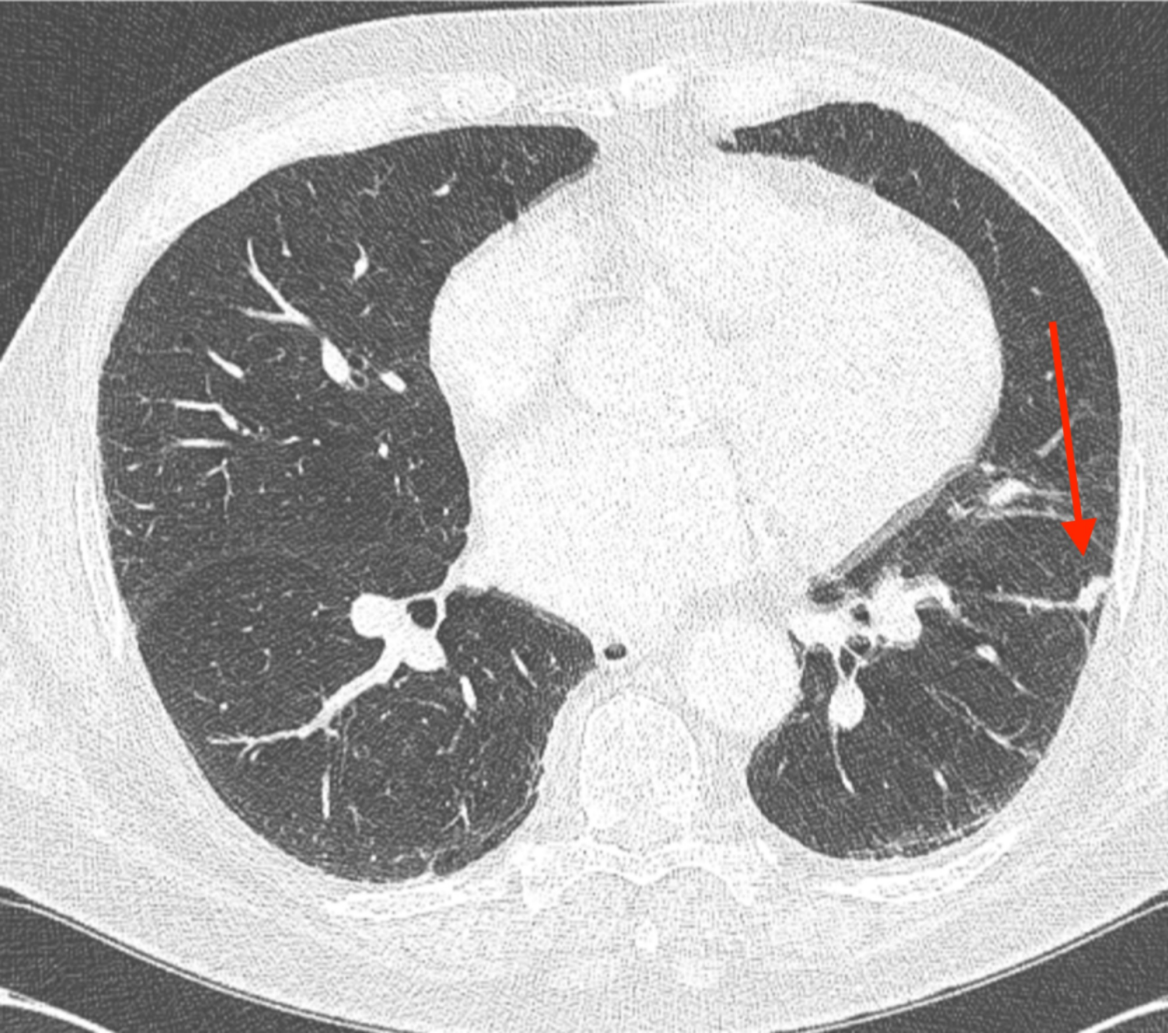
Three months later, the nodule was essentially stable at 1.2 x 0.7 cm; scans were then moved to a schedule of every six months

**Figure 14:**
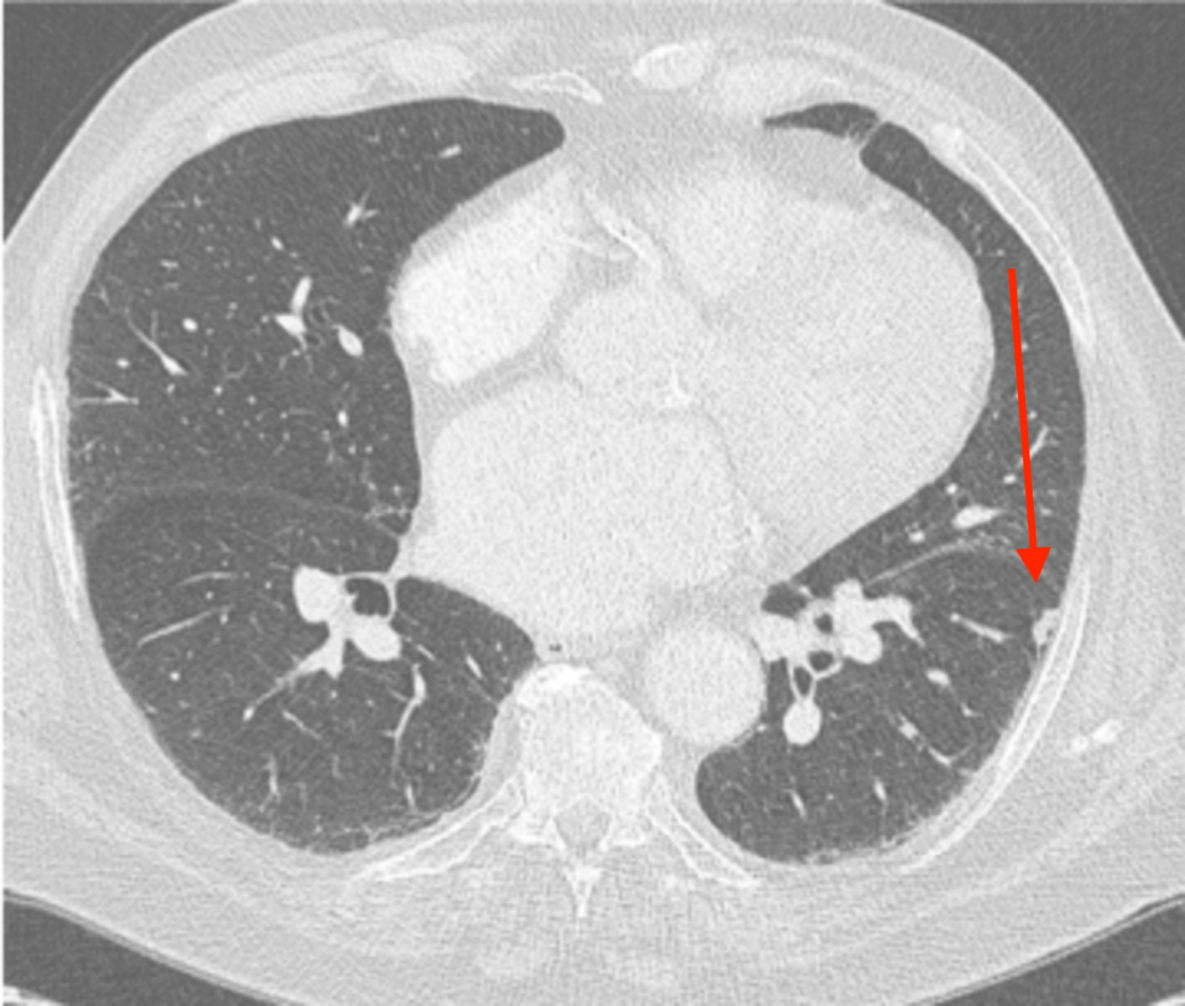
The subsequent scan performed 14 months post-biopsy revealed a residual 1.0 x 0.5 cm nodule in the left lower lobe

An additional surveillance CT performed at a three-month interval revealed a further reduction in the size of the left lower lobe lung mass from 2.2 cm to 1.6 cm. The patient elected to continue close monitoring. Two additional surveillance CT scans were obtained at three-month intervals, followed by an additional scan at a six-month interval, with continued diminution of the biopsy-proven left lower lobe tumor. On the most recent imaging, the residual tumor measured 1.0 cm by 0.5 cm, with no evidence of active disease now 14 months since the cytologic diagnosis. The radiographic evolution of his untreated, biopsy-proven tumor is shown in Figures 10-14.

## Discussion

Lung cancer is the second leading cause of cancer in both men and women in the United States. It is estimated that 200,00 new cases will arise in men and women in the United States accounting for 14% of new cancers5. Lung cancer accounts for 25% of cancer fatalities and has among the lowest survival rates [5].

Spontaneous regression of malignant tumors refers to partial or complete disappearance of tumors in the setting of inadequate or absence of therapy. This phenomenon was first defined by Everson and Cole in 1956 [6]; however, cases had been reported significantly earlier. A famous historical example of spontaneous tumor regression is that of Saint Peregrine [7]. Peregrine Laziosi (1265-1345) was a priest who developed a large ulcerative growth on his tibia in his 60s. This lesion was diagnosed as cancer, and it also appeared to be infected at the time. He was scheduled to undergo amputation, but, by the time of his scheduled operation, the tumor had disappeared and never recurred. 

The etiology of spontaneous tumor regression has not been clearly elucidated, but an autoimmune activating process has been suggested from reported cases in the literature, which often occur following inflammation, infection, surgery, or biopsy. Cole and Everson associate 40% of their case of spontaneous regression to operative trauma. In a review of cancer vaccinations, Butterfield discusses how tumor ablation could be a type of “cancer vaccine [8].”

This hypothesis was tested by Dr. Coley in the late 1890s. He first observed spontaneous regression of an inoperable neck sarcoma in a German immigrant patient who suffered erysipelas at the surgical site. After the infection transpired, the residual sarcoma disappeared and his wound healed rapidly. The patient remained disease free for seven years after. This particular case sparked the development of “Coley’s toxin”, a vaccine mixture of killed Streptococcus pyogenes and Serratia marcescens, to stimulate an inflammatory response.

We identified ten articles describing spontaneously regressing NSCLC between 1997 and 2018 in the published English language literature. In a similar case, Ogawa et al. describe a patient with metastatic poorly differentiated NSCLC whose tumors regressed following biopsy of a cervical lymph node metastasis and the primary lesion [9]. The author postulated that the invasive procedure triggered an immunologic stimulus leading to subsequent regression. More recently, Ariza-Prota et al. also reported a case of spontaneous regression following biopsy in a patient with metastatic squamous NSCLC [10]. 

One unusual case described by Kappauf et al. presented as metastatic bronchogenic cancer with metastasis to the abdominal wall [11]. The cancer underwent complete remission after biopsy, without febrile infection or conventional cancer treatment, following an excisional biopsy with incomplete resection. It was documented as a rare event, as there are few cases that document spontaneous regression following metastasis.

A number of the reported cases have involved spontaneous regression following biopsy, and investigators have postulated an association between regression with a potential trauma-mediated immunologic response leading to tumor regression. In an era where advances in immunotherapy have altered the treatment landscape for NSCLC, the possibility of inducing anti-tumor immune response through a trauma, such as a biopsy, is of particular interest. Many ongoing clinical trials evaluate the incorporation of radiation with immunotherapy, hypothesizing that the local immune response generated by radiation many enhance response to immunotherapy agents [12]. Cases of spontaneous regression following biopsy suggest other forms of local inflammatory response, including tumor manipulation or biopsy, could also be explored in tandem with immunotherapy.

Our patient had never received treatment with a systemic immunotherapy agent. However, he had a long history of treatment with intravesicular BCG for multiple recurrent, low grade, non-invasive bladder cancer. His most recent series of weekly instillations was completed approximately one month prior to his lung biopsy. Although intravesicular BCG involves local application of an immunomodulatory agent, systemic side effects, including inflammatory side effects, are well-established with this agent. Reports have shown up to a 0.7% rate of pneumonitis and 2.9% rate of fevers following intravesicular BCG [13]. Although our patient did not have any systemic clinical symptoms following instillation, the proximity to his tumor biopsy and subsequent tumor regression is intriguing and suggests a possible association. 

## Conclusions

Spontaneous regression of NSCLC is an uncommon occurrence. The underlying mechanism is poorly understood. Continued evaluation of patients with regressing NSCLC and a collection of samples could help in the understanding of its underlying mechanism. Better understanding the likelihood and course of spontaneously regressing cancers could inform physicians and patients about the varied and unpredictable course of cancer.
